# Complications Encountered with Non-adherence to Regular Follow-up in the Long-term Medical Management of Hyperparathyroidism

**DOI:** 10.7759/cureus.3966

**Published:** 2019-01-26

**Authors:** Ronak Raheja, Erica B Wolfish, Jill M Huded

**Affiliations:** 1 Internal Medicine, Kempegowda Institute of Medical Sciences, Bangalore, IND; 2 Family Medicine, Case Western Reserve University School of Medicine, Cleveland, USA; 3 Internal Medicine, Louis Stokes Cleveland VA Medical Center, Cleveland, USA

**Keywords:** primary hyperparathyroidism, non adherence, long-term follow-up, complications

## Abstract

Primary hyperparathyroidism is the third most commonly encountered endocrine disorder after diabetes and thyroid diseases. There has been a constant debate between medical and surgical management of the disorder. Guidelines clearly indicate surgical management over medical management in symptomatic patients and asymptomatic patients below 50 years of age. The problem is identification of symptoms can be difficult as there is a large overlap in the presentation of symptomatic and asymptomatic patients. Here, a 74-year-old veteran presented with scrotal edema and a perineal abscess. He had urinary incontinence secondary to urological procedures which were done for nephrolithiasis, which were detected incidentally on imaging. He had multiple vertebral compression fractures and required referral to neurosurgery. He had worsening renal function and cognitive impairment. On review of his medical records he was found to have a long-standing history of medically managed hyperparathyroidism, which was complicated due to non-compliance to follow-up outpatient visits. He constantly declined elective parathyroidectomy but unfortunately had to undergo several other invasive procedures with multiple hospital visits due to the complications of hyperparathyroidism. Safe medical management of hyperparathyroidism requires a religious follow-up and compliance to outpatient visits. He was started on Denosumab which we attribute to be contributory to his skin infections although evidence to support the same is insufficient.

## Introduction

Primary hyperparathyroidism is commonly encountered in the hospital setting and very often patients that are being medically managed become non-adherent to routine health care and suffer drastic complications which could have been easily prevented by a simple surgery. Very often patients get lost due to interdepartmental referrals and end up suffering due to inadequate information and health education. Our goal is to highlight the complications encountered in non-adherence to healthcare in the long-term medical management of hyperparathyroidism.

## Case presentation

A 74-year-old male with past medical history of atrial fibrillation, hypertension, dyslipidemia, and benign prostatic hypertrophy presented to the emergency department with testicular swelling and pain for four days. Physical exam was notable for an abscess on the inner gluteal fold of the perineal region that was spontaneously draining serosanguinous output. His left hemi-scrotum appeared erythematous and indurated without overt signs of cellulitis. There was mild tenderness to palpation of the scrotal area although no crepitus was felt in the thighs or scrotum. He also endorsed blood-tinged drainage from the wound for several weeks. He was admitted for the management of his scrotal wound. He was evaluated by urology and general surgery who had low suspicion for Fournier’s gangrene. Medical records demonstrated several urological procedures for ureterolithiasis and nephrolithiasis in the few months prior to this admission. The procedures included a bilateral ureteroscopy with laser lithotripsy followed by stone removal, stricturotomy, and placement of ureteral stents to prevent the progression of hydronephrosis. He had developed iatrogenic urinary incontinence after the procedures and had an indwelling Foley catheter inserted in view of incontinence.

**Figure 1:**
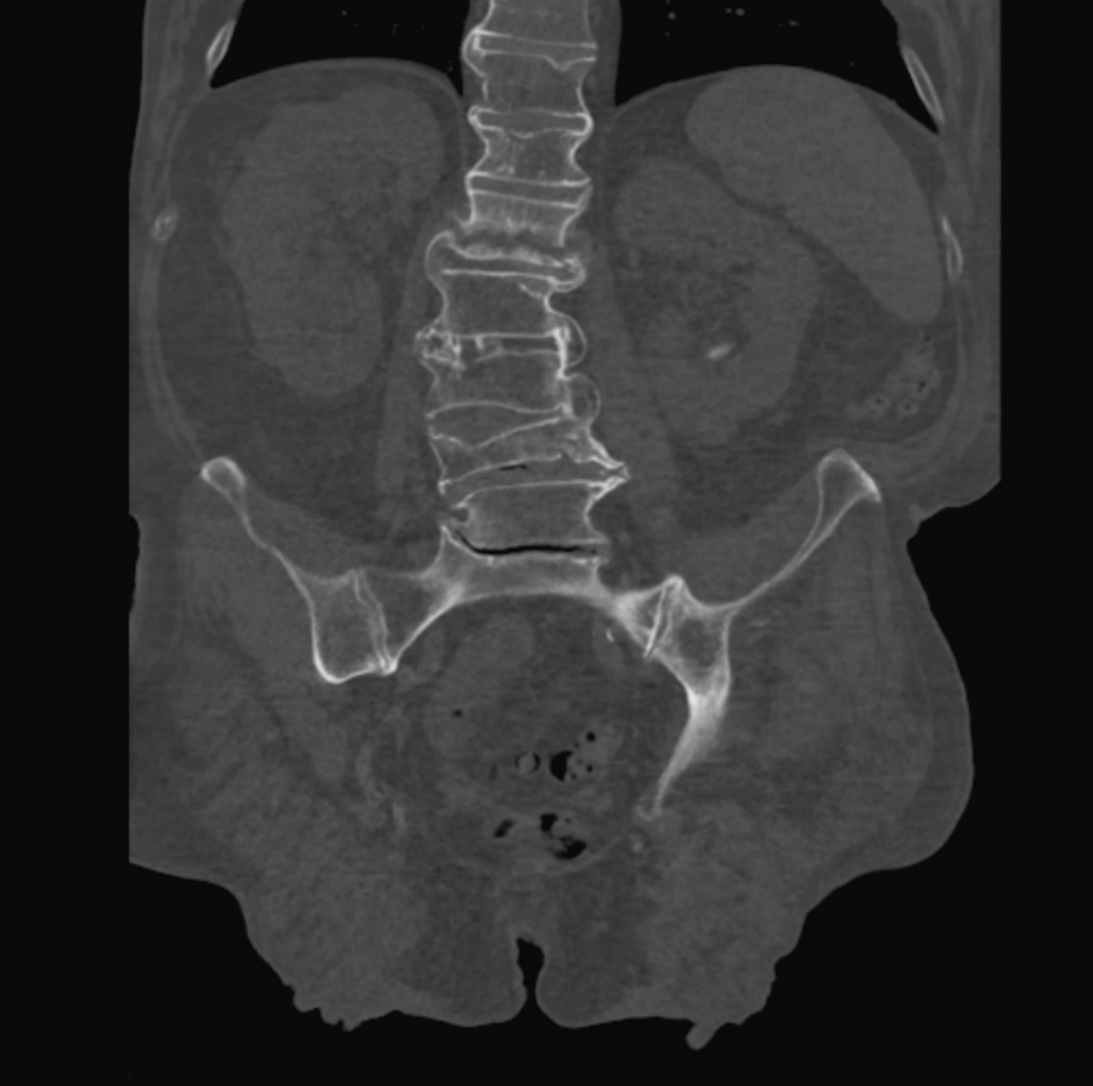
Computed tomography (CT) scan of the patient showed severe fractures of the spine.

He also reported a fall at home three weeks ago, following which he developed persistent back pain but did nothing about it. On our evaluation the computed tomography (CT) scan of the spine (Figure 1) revealed a recent L1 vertebral compression fracture with sclerosis and slightly ill-defined margins with additional lumbar spine compression fractures of T12 , L3 and L4 with several other multilevel degenerative changes in the lumbar spine. The patient also reported a gradual drop in height from 6 feet 3 inches to 5 feet 9 inches over the course of many years.

Upon admission to our inpatient department, review of his CT findings was out of proportion to age-related bone changes which led us to go back and do an extensive chart review which revealed that the patient had a long-concealed history of hyperparathyroidism, which was diagnosed incidentally from a routine blood draw which demonstrated hypercalcemia 12 years ago. He was initially referred by his primary care physician to endocrinology who strongly recommended surgical removal of the adenoma. However, the patient declined surgical intervention because he felt he was asymptomatic.The patient instead opted for medical management of his hyperparathyroidism and was started on long-term alendronate therapy by his primary care physician for approximately 10 years. Home care records demonstrate that he was non-adherent to routine follow-up and was predominantly home bound with minimal activity and minimal sunlight exposure. His laboratory records persistently demonstrated hypercalcemia and increased parathyroid hormone levels. At current admission his calcium was 8.9 mg/dl, parathyroid hormone was at 102 ng/L, vitamin D was 24 ng/ml and alkaline phosphatase was 75 IU/L.

Upon directed evaluation in the inpatient setting we found that along with nephrolithiasis and bone changes he had also developed other classical signs such as neurocognitive changes such as hallucinations of men standing in the air with food carts. He continued to decline parathyroidectomy despite worsening of his disease. His 10-year-long alendronate course was recently replaced by denosumab in view of treatment failure. Alternatively at the current admission denosumab was discontinued in view of increased risk of infections and he was started on cinacalcet therapy and intravenous antibiotic and was referred to the neurosurgery unit for the management of his chronic unstable L1 vertebral fracture.

## Discussion

Why this case report?

This is interesting to discuss because primary hyperparathyroidism is the third most commonly encountered endocrinopathy after diabetes and thyroid diseases [1].

Even the latest guidelines have not been able to successfully differentiate between asymptomatic and mildly symptomatic disease. It is clear to most medical practitioners that patients with symptomatic hyperparathyroidism should have surgery as surgery is the only definitive therapy [2]. The problem is that it is often difficult to differentiate between symptomatic and asymptomatic hyperparathyroidism, as the symptoms may often be vague and difficult to quantify. Only approximately one out five patients are clearly symptomatic with demarcated kidney stones, bone changes or with neuromuscular signs or symptoms [3]. The classical presentation of neuromuscular signs associated with hypercalcemia is usually subjective, and often difficult to categorize as symptomatic or not.

What are the indications for surgery?

Based on the Fourth International Workshop on Asymptomatic Primary Hyperparathyroidism guidelines patients need to meet only one of these four criteria to be considered for surgery as shown in Table 1.

**Table 1:** Fourth International Workshop on Asymptomatic Primary Hyperparathyroidism guidelines to be considered for parathyroid surgery. CT: Computed tomography; MRI: Magnetic resonance imaging; GFR: Glomerular filtration rate.

1) Serum calcium	Concentration of 1 mg/dl or more above the upper limit of normal
2) Skeletal indications	a) Bone density at any one site such as the hip, lumbar spine or distal radius that is 2.5 times below peak bone mass
	b) Previous asymptomatic vertebral fracture (by radiograph, CT or MRI)
3) Renal indications	a) Estimated GFR less than 60 ml/min
	b) 24 hours urinary calcium > 400 mg/day
	c) Nephrolithiasis or nephrocalcinosis on imaging
4) Age	Completely asymptomatic and below 50 years of age

What is the biggest factor to consider in medically managed patients?

Our patient demonstrated 10 years of non-adherence and the single most important factor to note in medically managed patients is adherence to regular monitoring for serum calcium, creatinine, estimated glomerular filtration rate (eGFR) annually and bone density (hip, spine, and forearm). Monitoring should be done every one or two years to prevent complications and if any indication of surgery arises despite any form of therapy we should opt for surgery to prevent worsening of the disease [2].

Complications Related to Bones in Hyperparathyroidism

Even in asymptomatic patients, there was a decreased cortical thickness observed as compared to trabecular bone which was usually spared; this effect was further pronounced with vitamin D deficiency [4, 5]. There have been several studies which show an increase in fracture risk in hyperparathyroidism. A decrease in bone mineral density has been established more prominently at cortical sites [6]. Therefore regular monitoring of vitamin D is indicated, and supplementation is indicated in those with vitamin D deficiency. Our patient was non-compliant therefore we could not supplement vitamin D regularly [7].

Management with Bisphosphonates

Studies have demonstrated bisphosphonates to be as efficient as surgery for the first two years [8]. Data is insufficient for the long-term (10 years) use of bisphosphonates without adequate follow-up in patients such as ours. Studies show a significant reduction of the incidence of nephrolithiasis in patients that have been surgically managed as compared to medically managed patients [9]. Based on these results, once someone has nephrolithiasis it is a clear indication for parathyroidectomy.

Indication of Cinacalcet

Once the indication for surgery is clear and the patient is a poor candidate for surgery he would have to be medically managed; the guidelines summary statement from the Fourth International Workshop recommends stopping bisphosphonates and starting cinacalcet at an initial dose of 30 mg twice daily and incrementally increasing the dose up to 90 mg three or four times a day [2].

Stones

Most stones in patients with primary hyperparathyroidism are composed of calcium oxalate [10]. Nephrolithiasis is seen in 15–20% of patients with primary hyperparathyroidism [11].

Apart from renal stones, what are the other renal manifestations associated with asymptomatic hyperparathyroidism?

Worsening of renal function was attributed to be directly related to increasing serum calcium levels which was related to increasing parathyroid hormone levels. This concept was demonstrated in a study which showed 17% of asymptomatic primary hyperparathyroidism patients showed a subclinical renal disease with estimated glomerular filtration being below 60 ml/min [12].

Psychic Groans

Neurocognitive behavioral patterns have been clearly demonstrated as more prevalent in patients with hyperparathyroidism, as compared to the general population [13]. Several studies have shown improvement in neurocognitive function post parathyroidectomy [14].

Cardiac Manifestations

Studies have shown an increased association between hyperparathyroidism and hypertension [15]. Some studies show an increase in left ventricular thickness and have demonstrated significant reduction of left ventricular mass post parathyroidectomy [16]. Some studies show an increased carotid intimal thickness with hyperparathyroidism [17].

## Conclusions

This patient initially declined a simple surgery but later on ended up having to undergo multiple invasive urological procedures. He also experienced severe spinal compression fractures which were ultimately followed by neurosurgery. Medical management of this disease is only feasible if the patient complies with yearly monitoring and regular follow-up. If a patient starts to develop complications, surgery should be performed to prevent progression of the disease. We suspect denosumab to be contributory to the development of his perianal abscess and its abrupt cessation could be contributing to the development of vertebral fractures although evidence to establish the same is insufficient and requires further evaluation.
